# Visualization and assessment of saccular duct and endolymphatic sinus

**DOI:** 10.3109/00016489.2010.533697

**Published:** 2011-03-03

**Authors:** Hideo Yamane, Masahiro Takayama, Kishiko Sunami, Hiramori Sakamoto, Toshio Imoto, Matti Anniko

**Affiliations:** 1 Department of Otorhinolaryngology, Osaka City University Graduate School of Medicine, Osaka, Japan; 2 Department of Otorhinolaryngology and Head & Neck Surgery, Uppsala University Hospital, Uppsala, Sweden

**Keywords:** Cochlea, saccule, bony groove, longitudinal flow, CT image, endolymphatic hydrops, endolymphatic duct, rendering

## Abstract

*Conclusion:* The saccular duct and endolymphatic sinus run in the bony groove, before reaching the orifice of the vestibular aqueduct. We first clinically visualized this sulciform groove using three-dimensional (3D) cone beam CT images. This strategy can be useful to assess the condition of the saccular duct and endolymphatic sinus concerning the longitudinal flow system of endolymph. *Objective:* To assess the saccular duct and endolymphatic sinus in the endolymphatic system in order to advance clinical studies on inner ear dysfunction. *Methods:* The sulciform groove of the saccular duct and endolymphatic sinus of human subjects was analyzed by cone beam CT and compared with that of a cadaver. *Results:* We could obtain reconstructed 3D CT images of the sulciform groove of the saccular duct and endolymphatic sinus using several CT window levels.

## Introduction

The saccule connects with two ducts, the reuniting and saccular ducts, which are respectively the paths of endolymph. These paths are important in the longitudinal flow theory [1], whereby the endolymph goes directly from the cochlea to the endolymphatic sac. This theory explains the cause of Meniere's disease, which exhibits endolymphatic hydrops [2,3]. From this viewpoint, the vestibular aqueduct that follows the saccular duct was initially clinically visualized and used as a diagnostic tool for Meniere's disease [4,5]. We also clinically analyzed the reuniting duct by visualizing it with three-dimensional computed tomography (3D CT) to diagnose Meniere's disease [6-8]. However, the saccular duct has not been clinically visualized. The reason is that the saccular duct is membranous, facing endolymph inside and peri-lymph outside, and so CT and MRI have limitations for visualizing it. The saccular duct becomes endolymphatic sinus running in the bony sulciform groove before ending in the vestibular aqueduct, which may be visualized in 3D CT images as a bony substance, like the bony groove of the reuniting duct (YT groove) in a previous study [6,7].

In the present study, we investigated whether the saccular duct and endolymphatic sinus could be demonstrated on clinical images using cone beam 3D CT.

## Material and methods

We investigated the saccular duct and endolymphatic sinus and their sulciform groove in the temporal bones of bilaterally healthy ears of 12 controls (6 males and 6 females; mean age 58.4 years, range 35-79 years), which had been included in a previous study [8] employing cone beam CT (3D Accuitomo; J. Morita Mfg Corp., Kyoto, Japan) and the temporal bone of a cadaver donated to our medical university of anatomy, with the consent of the deceased and our university, to compare the findings in human subjects with those in the cadaver.

CT images were investigated using cone beam CT to obtain images under the following conditions: 80 kV, 6 mA, voxel 0.125 × 0.125 × 0.125 mm, slice thickness 0.5 mm. CT images of this region were taken as a column with a diameter and height of 6 cm. Reconstructed 3D images of the inner ear were obtained using rendering software (IVIEW; J. Morita Mfg Corp.) using a perspective view with a viewing angle of 15° and a 0.25 mm voxel (0.25 × 0.25 × 0.25 mm).

To obtain 3D CT images of the saccular duct and endolymphatic sinus in accordance with cadaver specimens, we adopted the landmarks reported in a previous study to reduce discrepancies due to rendering effect [7]. 3D CT images are manipulated so that the saccule, YT groove, and vestibular portion of the posterior semicircular canal and ridge of the cochlea just sloping down to the vestibule in front are all viewed in one frame. With this view, the bony sulciform groove of the saccular duct and endolymphatic sinus is visible. Then, this image can be manipulated through pitch or yaw to yield a view from directly above the sulciform groove to decrease the artifact by rendering effects. Also, several CT window levels were used in one view to visualize the groove from the surrounding architecture. Approval was obtained from the ethics committee of Osaka City University Graduate School of Medicine.

## Results

**Figure 1:**
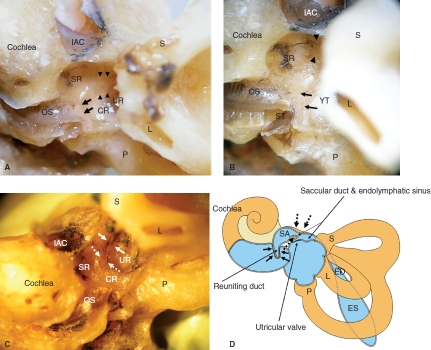
Macroscopic findings of the vestibule in the cadaver (left ear). (A) Membranous composition of the saccule and its intact connecting duct. Arrows show the reuniting duct, and arrowheads show the saccular duct and endolymphatic sinus. (B) Membranous compositions of the saccule and reuniting duct were removed. Arrows show the YT groove of the reuniting duct. Arrowheads, saccular duct. (C) The sulciform groove of the saccular duct (white dashed arrows) and endolymphatic sinus (white arrows) reach the orifice of the vestibular aqueduct. (D) Schematic view of the YT groove (bold arrows) of the reuniting duct and sulciform groove (dashed arrows) of the saccular duct and endolymphatic sinus. CR, cochlear recess; ED, endolymphatic duct; ES, endolymphatic sac; IAC, internal auditory canal; L, lateral semicircular canal; OS, osseous spiral lamina; P, posterior semicircular canal; S, superior semicircular canal; SA, saccule; SR, saccular recess; UR, utricular recess.

**Figure 2:**
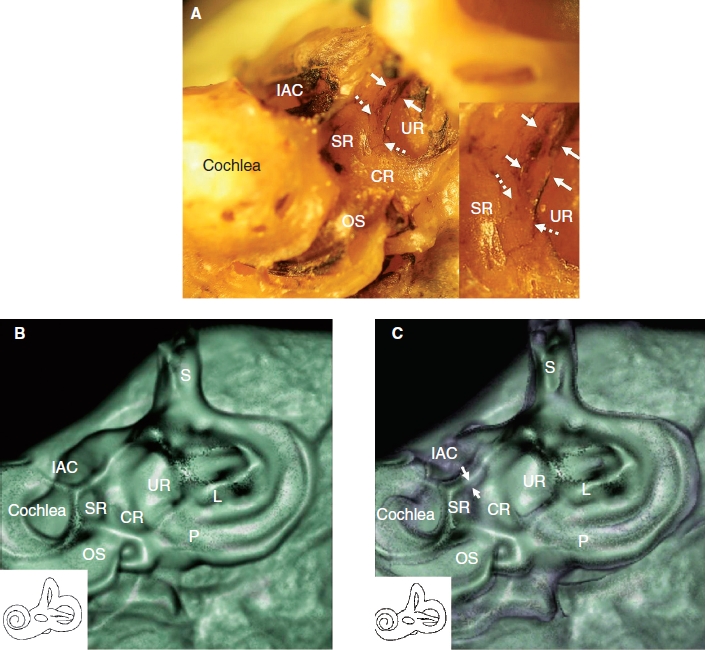
Direction of CT examination and CT findings in the cadaver. (A) This position of the temporal bone is suitable to obtain more accurate CT images of the saccule, saccular duct, and endolymphatic sinus. Note that the remnant membranous labyrinth, shown in black, is visible. Insert: Higher magnification of the groove of the saccular duct (white dashed arrows) and endolymphatic sinus (white arrows). (B) The sulciform groove-like 3D CT image of the saccular duct and endolymphatic sinus of the same specimen as in (A), which was examined in the same direction as in (A) with a bone CT window level. (C) The same specimen as in (A) with CT window levels of bone and soft tissue. The membranous composition of the saccular duct and endolymphatic sinus in purple (white arrows) are surrounded by the artificial shadow of a rendering effect. The details of the aspects are consistent with macroscopic specimens. Purple indicates soft tissue. CR, cochlear recess; IAC, internal auditory canal; L, lateral semicircular canal; OS, osseous spiral lamina; P, posterior semicircular canal; S, superior semicircular canal; SR, saccular recess; UR, utricular recess.

A sulciform groove from the saccular fossa to the orifice of the vestibular aqueduct in which the saccular duct and endolymphatic sinus run could be localized in the cadaver specimen (Figure 1A-D). The sulciform groove of the endolymphatic sinus was distinct but that of the saccular duct was unclear (Figures 1C and 2A). Employing a direction in which the saccule and saccular duct and endolymphatic sinus can be seen from directly above, the groove-like 3D CT image of the saccular duct and endolymphatic sinus were consistent with macroscopic findings (Figure 2A-C). This groove-like image was made up of two colors when CT was imaged with these bone and soft tissue CT window levels (Figure 2C). Further, this image was consistent with macroscopic cadaveric findings because there was a torn membranous labyrinth around the groove that could be imaged as soft tissue by CT.

**Figure 3:**
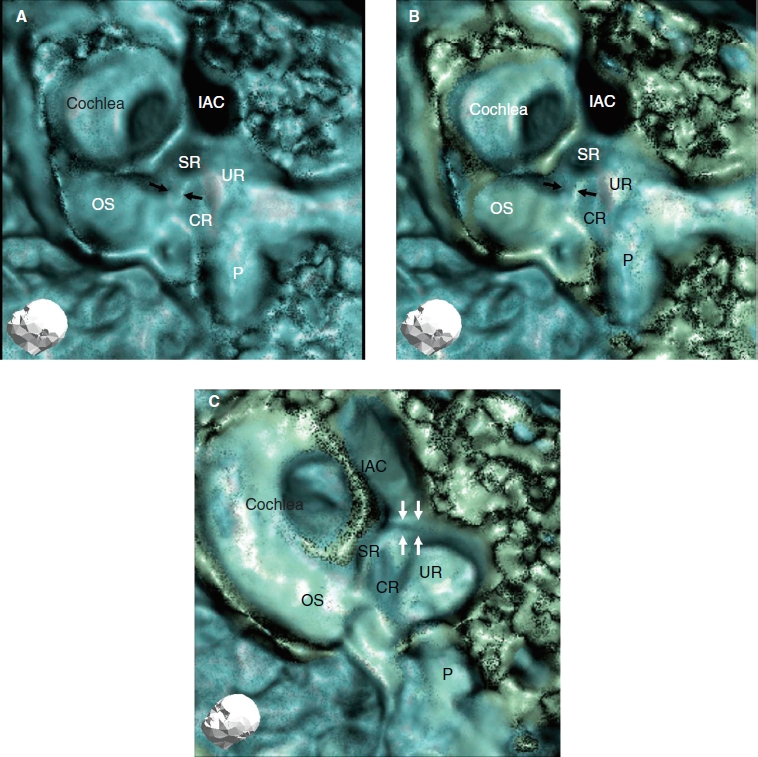
Findings of a representative 3D CT image of a healthy human subject (left ear). (A) With low-level bone CT window (blue color). (B) With low - and high- level bone CT windows (blue and green color). (C) The image shows a view from directly above the sulciform groove with the same CT window level as in (B). A more accurate image of the sulciform groove of the saccular duct and endolymphatic sinus can be judged by referring to the CT images in (A, B, and C) The method in (C) is suitable for assessment of the sulciform groove of the saccular duct and endolymphatic sinus. With two different bone densities the CT view manifests more three-dimensionally. The cochlear lateral wall and basal portion of the osseous spiral lamina in green seem to be denser in bone density than the vestibule and the apex of the cochlea in blue. CR, cochlear recess; IAC, internal auditory canal; OS, osseous spiral lamina; P, posterior semicircular canal; SR, saccular recess; UR, utricular recess. Black arrows, YT groove; white arrows, the sulciform groove of the saccular duct and endolymphatic sinus.

**Figure 4:**
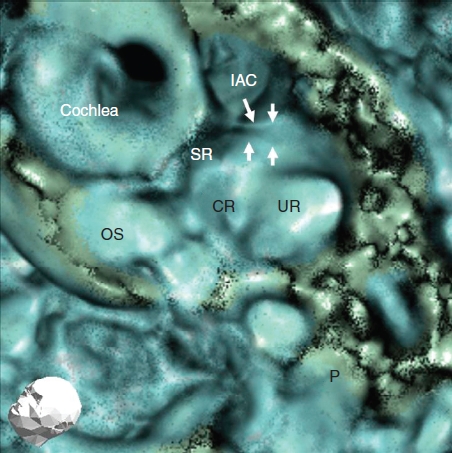
Findings of other representative 3D CT images in a healthy human subject (left ear). CR, cochlear recess; IAC, internal auditory canal; OS, osseous spiral lamina; P, posterior semicircular canal; SR, saccular recess; UR, utricular recess. White arrows, the sulciform groove of the saccular duct and endolymphatic sinus.

Groove-like 3D CT images of the saccular duct and endolymphatic sinus were obtained in all 12 human subjects. The aspects of the groove were similar among the 12 healthy subjects (Figures 3 and 4).

The images of the groove reconstructed using several CT window levels such as a high or low density of bone revealed more accurate images (Figure 3B and C), especially the lumen of the groove, which was more manifested three-dimensionally compared with single CT window levels (Figure 3A-C).

## Discussion

The vestibular aqueduct has been assessed in relation to the etiology of Meniere's disease, where the endolymphatic duct runs and reaches the endolymphatic sac. This clinical strategy is based on the fact that idiopathic endolymphatic hydrops, a pathological entity of Meniere's disease, is caused by the blockage of endolymph in the endolymphatic duct or sac [9,10].

However, there are few reports on the saccular duct and endolymphatic sinus despite these sites and the vestibular aqueduct being directly connected.

Most saccular duct and endolymphatic sinus studies are limited to animal experiments or specimens of human temporal bone [3,10], because the saccular duct and endolymphatic sinus are too small and membranous to assess clinically. The saccular duct and endolymphatic sinus are not a bony groove themselves but run in the bony groove, which could be visualized clinically using 3D CT. When the saccular duct or endolymphatic sinus is involved as a lesion in inner ear disease, their bony grooves may reflect the lesional effect. A similar strategy was adopted to assess the reuniting duct to visualize the YT groove in a previous study [6,7].

The rendering strategy for 3D CT analysis sometimes leads to a misunderstanding [7]. The CT image of the cadaver with a double CT window level such as bone and soft tissue showed the groove and its circumscribing torn membranous labyrinth, in which the image was consistent with the macroscopic cadaver-based findings. Therefore, we judged that our strategy could markedly reduce the negative effect of rendering. As precise differentiation of the saccular duct from the endolymphatic sinus was difficult when employing 3D CT images, we assessed both portions as one unit in the present study.

In human cases, the CT images of the sulciform groove of the saccular duct and endolymphatic sinus from directly above using different bone CT window levels on one image were consistent with cadaver specimen findings, and much more informative than the images employing a single bone CT level.

We previously reported the importance of assessing the reuniting duct, which is frequently involved in Meniere's lesions, and dislodged otoconia from the saccule into the reuniting duct [8]. As the saccular duct directly connects with the saccule, we cannot deny the possibility that dislodged otoconia from the saccule may enter the saccular duct or endolymphatic sinus in a similar manner to that in the reuniting duct.

Investigating the saccular duct and endolymphatic sinus will provide novel information regarding inner ear diseases caused by longitudinal flow disturbances of endolymph, especially Meniere's disease. We will also further investigate the role of these architectures.
